# Wallerian degeneration in cervical spinal cord tracts is commonly seen in routine T2-weighted MRI after traumatic spinal cord injury and is associated with impairment in a retrospective study

**DOI:** 10.1007/s00330-020-07388-2

**Published:** 2020-10-30

**Authors:** Tim Fischer, Christoph Stern, Patrick Freund, Martin Schubert, Reto Sutter

**Affiliations:** 1 Department of Radiology, University Hospital Balgrist, Forchstrasse 340, 8008 Zurich, Switzerland; 2 Spinal Cord Injury Center, University Hospital Balgrist, Forchstrasse 340, 8008 Zurich, Switzerland

**Keywords:** Trauma, Spinal cord, Wallerian degeneration, Magnetic resonance imaging, Observational study

## Abstract

**Objectives:**

Wallerian degeneration (WD) is a well-known process after nerve injury. In this study, occurrence of remote intramedullary signal changes, consistent with WD, and its correlation with clinical and neurophysiological impairment were assessed after traumatic spinal cord injury (tSCI).

**Methods:**

In 35 patients with tSCI, WD was evaluated by two radiologists on T2-weighted images of serial routine MRI examinations of the cervical spine. Dorsal column (DC), lateral corticospinal tract (CS), and lateral spinothalamic tract (ST) were the analyzed anatomical regions. Impairment scoring according to the American Spinal Injury Association Impairment Scale (AIS, A–D) as well as a scoring system (0–4 points) for motor evoked potential (MEP) and sensory evoked potential (SEP) was included. Mann-Whitney *U* test was used to test for differences.

**Results:**

WD in the DC occurred in 71.4% (*n* = 25), in the CS in 57.1% (*n* = 20), and in 37.1% (*n* = 13) in the ST. With WD present, AIS grades were worse for all tracts. DC: median AIS B vs D, *p* < 0.001; CS: B vs D, *p* = 0.016; and ST: B vs D, *p* = 0.015. More pathological MEP scores correlated with WD in the DC (median score 0 vs 3, *p* < 0.001) and in the CS (0 vs 2, *p* = 0.032). SEP scores were lower with WD in the DC only (1 vs 2, *p* = 0.031).

**Conclusions:**

WD can be detected on T2-weighted scans in the majority of cervical spinal cord injury patients and should be considered as a direct effect of the trauma. When observed, it is associated with higher degree of impairment.

**Key Points:**

*• Wallerian degeneration is commonly seen in routine MRI after traumatic spinal cord injury.*

*• Wallerian degeneration is visible in the anatomical regions of the dorsal column, the lateral corticospinal tract, and the lateral spinothalamic tract.*

*• Presence of Wallerian degeneration is associated with higher degree of impairment.*

## Introduction

Wallerian degeneration (WD) is a well-known phenomenon and describes disintegration of axons and myelin sheaths after the connection with the cell body is interrupted [1]. Although originally only antegrade degeneration was described in 1850 by Waller [2], there is evidence for a common mechanism in antegrade and retrograde degeneration [3]. In this article, both antegrade and retrograde degeneration is referred to as WD. Common causes include cerebral infarction, WD is less commonly seen in hemorrhage, neoplasm, surgery, epilepsy, and white matter disease [4, 5].

WD can be identified on routine imaging as a T2-weighted (T2w) hyperintense signal visible after 10–14 weeks with subsequent shrinkage over several years [6].

On the histopathologic level, the process evolves in several stages [7]. Histopathologic changes have been shown to precede visible changes on MRI in the cervical spinal cord and WD begins as early as 8 days after injury [8]. Few studies have addressed tract-specific WD in the spinal cord with focus on the dorsal column (DC) and the lateral corticospinal tract (CS) using advanced MRI methods such as high angular diffusion-weighted imaging, magnetization transfer, diffusion tensor imaging, or fractional anisotropy [1, 4, 9–14]. However, it is not clear whether WD can commonly be detected on the clinical MRI examinations that the patients with traumatic spinal cord injury (tSCI) receive as part of standard care.

To our knowledge, there is only little data in form of two case reports about WD in the setting of a routine clinical MRI [5, 15]. This is the first study that examines occurrence of WD in routine MRI after traumatic spinal cord injury (tSCI) and correlation with clinical deficits.

## Methods

### Patients

This retrospective study included patients with tSCI that underwent MRI of the cervical spine from January 2004 until August 2019 and was approved by the local ethics committee. A total of 61 patients with tSCI were initially included with at least 3 follow-up MRI examinations (time interval between trauma and most recent MRI examination being at least 1 year) who were referred to our institution for rehabilitation. Scoring of trauma severity was done according to American Spinal Injury Association Impairment Scale (AIS) which is based on clinical examination according to the International Standards for Neurological Classification of Spinal Cord Injury [16]. These clinical and additional neurophysiological assessments by sensory (SEP) and motor evoked potentials (MEP) were performed between 2 and 20 days after trauma.

### Study design

**Fig. 1:**
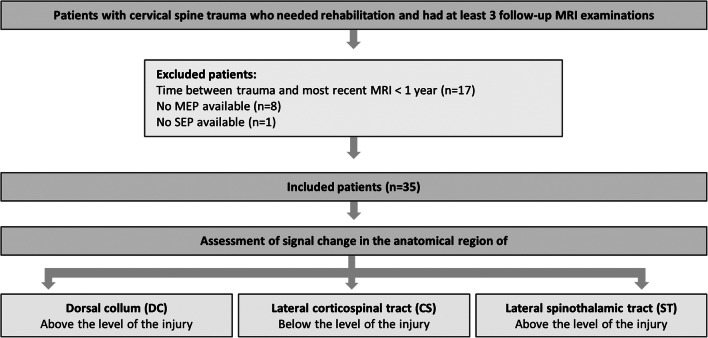
Flow diagram of the study design and included patients. All patients were evaluated for signal intensity change consistent with Wallerian degeneration (WD) in the region of the dorsal column (DC), the lateral corticospinal tract (CS), and the lateral spinothalamic tract (ST)

The study design is presented in Fig. 1. Cervical spine MRIs were evaluated for T2w signal hyperintensity in the anatomical region of specific tracts, consistent with WD. Signal intensity change was assessed in the anatomical region of the dorsal column (DC), the lateral corticospinal tract (CS), and the lateral spinothalamic tract (ST) [17–19]. T2w signal hyperintensity was evaluated in at least three successive MRI examinations after trauma. If a specific hyperintense signal was visible in one or more MRI examinations, the signal was evaluated as positive, even if a subsequent MRI was read negative for signal change in the same location. When no signal change was visible in all available MRI examinations for at least 1 year, this patient was evaluated negative for WD.

### Imaging and image evaluation

Most patients underwent MRI of the cervical spine on a 3T Magnetom Skyra fit system (Siemens Healthineers). Image evaluation was performed on sagittal and axial T2w turbo spin-echo images. Following settings were used: axial: repetition time (TR) 5510 ms, echo time (TE) 93 ms, echo train length (ETL) 16, field of view: (FOV) 160 × 160 mm, matrix 320 × 320, slice thickness 3 mm, spacing 3.6 mm. Sagittal: repetition time (TR) 3760 ms, echo time (TE) 87 ms, echo train length (ETL) 17, field of view (FOV) 220 × 220 mm, matrix 384 × 384, slice thickness 2.5 mm, spacing 2.75 mm. In cases with significant metal artifacts or before the 3T system was installed, imaging was done on a 1.5T Avanto fit system (Siemens Healthineers), using the following settings: Axial: repetition time (TR) 3390 ms, echo time (TE) 112 ms, echo train length (ETL) 15, field of view (FOV) 200 × 200 mm, matrix 320 × 320, slice thickness 3 mm, spacing 3.5 mm. Sagittal: repetition time (TR) 3440 ms, echo time (TE) 107 ms, echo train length (ETL) 16, field of view (FOV) 240 × 240mm, matrix 512 × 512, slice thickness 2.5 mm, spacing 2.75 mm. In both systems, a standard radiofrequency neck coil (20 channels) is used. Our 1.5T and 3T systems received a technical update (Verio to Skyra fit and Avanto to Avanto fit) in 2013. Systems originally were installed in 2004 (Avanto) and 2010 (Verio).

Axial T2w images had to cover at least two segments above and below the injury. Two fellowship trained radiologists with 6 and 7 years of experience in radiology and at least 1 year exclusively in neuroradiology evaluated all patients on our institution’s Merlin PACS (Phoenix-PACS GmbH).

First, in the trauma MRI, the level of the injury and sagittal expansion were evaluated on sagittal T2w images; clinical information was withheld from the readers. Transverse cord involvement was evaluated, using the Brain and Spinal Injury Center score (BASIC) grade 0–4. Grade 0: No appreciable cord signal abnormality; grade 1: Intramedullary T2w hyperintensity is approximately confined to central gray matter; grade 2: Intramedullary T2w hyperintensity extends beyond expected gray matter margin to involve spinal white matter, but does not involve entire transverse extent of the spinal cord; grade 3: intramedullary T2w hyperintensity involves entire transverse extent of spinal cord; grade 4: Grade 3 injury plus discrete T2w hypointense foci, consistent with macrohemorrhage [20].

**Fig. 2:**
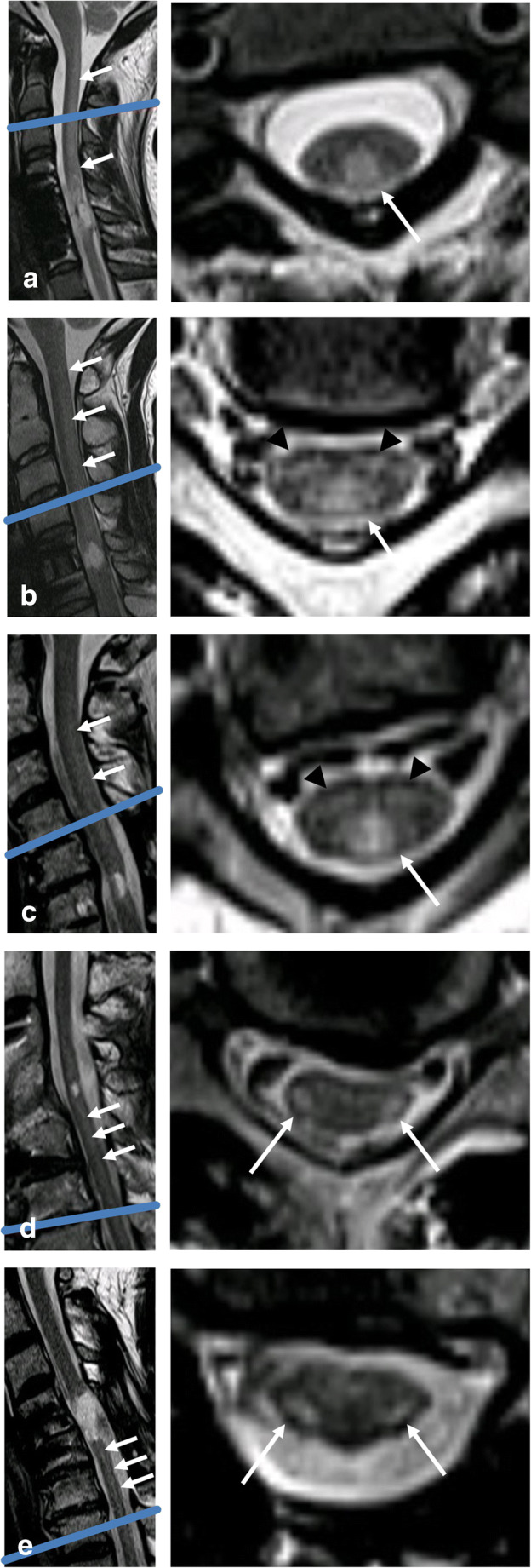
Sagittal and axial T2w MRI of the cervical spine of five different patients demonstrating examples of signal intensity change consistent with Wallerian degeneration (WD) in the dorsal column (DC), the lateral corticospinal tract (CS), and the lateral spinothalamic tract (ST). Axial plane is indicated by the blue line on the corresponding sagittal image. **a** A 19-year-old patient 348 days after motor vehicle accident (MVA) demonstrating WD in the DC on axial images (white arrow). On median, sagittal imaging, WD (white arrows) can be appreciated from the injury upwards to the medulla oblongata. **b** A 22-year-old patient 109 days after fall demonstrates WD in the DC on axial and median sagittal imaging (white arrows). In the anterior cord, bilateral WD in the ST is seen (black arrowheads). **c** A 60-year-old patient 111 days after surfing accident also demonstrates WD in the DC (white arrow) and ST (black arrowheads) on axial imaging. On median sagittal imaging, faint WD in the DC is seen (white arrows). **d** A 50-year-old patient 323 days after MVA demonstrates WD in the CS (white arrows) on axial imaging. On paramedian sagittal imaging, faint WD in the CS from the injury downwards can be appreciated (white arrows). **e** A 55-year-old patient 216 days after fall demonstrates WD in the CS on axial and paramedian sagittal imaging (white arrows)

In all available follow-up examinations, tract-specific signal change was evaluated without any clinical information. First, the level of the injury was determined on sagittal T2w. Evaluation of the axial T2w consisted of evaluation for (1) T2w hyperintense signal change in the region of the dorsal column (DC) one level above the level of the injury, (2) T2w hyperintense signal change in the region of the lateral corticospinal tract (CS) one level below the level of the injury, and (3) T2w hyperintense change in the region of the lateral spinothalamic tract (ST) one above the level of the injury. Signal abnormality was read as present or absent. Examples for signal abnormality in each tract are shown in Fig. 2.

### Clinical assessments

Severity assessment of spinal cord injuries (SCI) was performed by trained rehabilitation specialists. Classification was performed based on standardized assessment of segmental motor and sensory testing according to ISNCSCI. Clinical assessments were done twice, 0 to 15 days after trauma and 16 to 40 days after trauma (stage very acute and acute 1 follow-up according to the European Multicenter Study about Spinal Cord Injury, EMSCI). This allowed to determine AIS grades which ranged from A to D (A: motor-sensory complete; B: motor complete, sensory incomplete; C: motor-sensory incomplete; and D: motor-sensory incomplete, majority of key muscles below the lesion show movement against gravity [21, 22]).

### Neurophysiological examination

In order to objectively assess long spinal tract integrity, trained technicians and experienced physicians performed independent neurophysiological tests of lumbar dermatomes according to clinical standards on certified electromyography machines [23]. Examination was done between 16 to 40 days after trauma (stage acute 1 follow-up according to the European Multicenter Study about Spinal Cord Injury, EMSCI). Technical setup and rating was done according to Hupp et al [24]. Tibial nerve stimulation led to recordings of somatosensory evoked potentials (SEP). Motor evoked potentials (MEP) were recorded bilaterally from anterior tibial muscles following transcranial magnetic stimulation of the corresponding cortical motor areas. Latency and amplitude readings were obtained according to the used standard [24]. After normalization latencies for body height, test results were converted to a simplified score as previously described [24]: motor and sensory potentials were rated with a maximum score of 2 for each side if evoked potentials were normal with respect to latency and amplitude, one if pathological in one of these aspects, and zero if missing. In this way for both MEP and SEP assessments, a maximum of 4 points (2 points each side) could be achieved.

### Statistics

Statistical analysis was performed on SPSS version 21.0 (IBM Corp). For continuous data, general descriptive statistics were reported as means and standard deviation (SD). Normal distribution of patient age was evaluated with the Kolmogorov-Smirnov test. For ordinal-scaled variables (AIS grades, SEP and MEP scoring), Mann-Whitney *U* test was used to test for significant differences. For statistical analysis, AIS grades A–D were transferred to numeric (0–3), and results were transferred back to AIS grades (A–D) and reported as such. For effect size (*r*), the Cohen classification was used [25]. For *r* > 0.1, the effect was considered weak. *R* > 0.3 reflected a medium and *r* > 0.5 a strong effect. In all tests, a *p* value of < 0.05 was considered to represent statistical significance. The two-way random effects intraclass correlation coefficient (ICC) was applied for inter-reader agreement, whereas ICC values > 0.5 were moderate, > 0.75 were considered good agreement, and > 0.9 as very good [26].

## Results

In 17 cases, the interval between trauma and the most recent MRI examination was less than 1 year and these cases were excluded. MEP was not available in 8 cases and SEP in 1 case that led to exclusion of another 9 patients, resulting in a final set of 35 patients. In the trauma MRI, ICC for lesion level, sagittal expansion, and BASIC score were good: (0.87), (0.88), and (0.85).

Signal abnormality in the DC was detected by both readers with 100% accordance, resulting in an ICC of 1. For the CS, ICC was moderate (0.54), and for the ST, ICC was good (0.88). Disagreements were solved by consensus reading.

Results for each patient, including demographic information, mechanism of injury, initial MRI findings after trauma, and visible WD in at least one out of three follow-up MRIs as well as clinical and neurophysiological assessments, are given in Table 1. Mean time between trauma and first imaging was 1.8 days (SD ± 2.07, minimum 0 days, maximum 8 days).

**Table 1:** Included patients with demographic, clinical, neurophysiological, and imaging features

Trauma	Age	Gender	Level	Sg. length	Basic	AIS	SEP	MEP	WD DC	WD CS	WD ST
Fall	80 years	F	C3	1	2	D	2	4	No	No	No
Fall from stairs	78 years	M	C4	1	2	D	2	3	No	Yes	No
Bicycle accident	43 years	M	C5	2	2	B	2	3	Yes	No	Yes
MWA	19 years	M	C5	3	4	A	0	0	Yes	Yes	No
MWA	50 years	M	C4	2	3	D	4	4	Yes	Yes	No
Skiing accident	73 years	M	C3	1	2	B	4	2	Yes	Yes	No
Skiing accident	41 years	M	C4	2	2	B	2	0	Yes	Yes	Yes
Bicycle accident	52 years	M	C4	2	2	C	0	2	No	No	No
Fall	30 years	M	C7	2	3	A	0	0	Yes	Yes	Yes
Parachute accident	35 years	F	C5	3	4	C	1	0	Yes	No	Yes
Diving accident	21 years	M	C4	2	2	A	0	0	Yes	No	No
Fall	78 years	M	CS	4	1	C	2	0	Yes	No	No
Diving accident	18 years	Sr	C6	5	4	A	0	0	Yes	Yes	Yes
Fall	56 years	M	C4	2	2	D	2	2	No	No	No
Fall	43 years	M	C6	3	3	A	0	0	Yes	Yes	Yes
MWA	48 years	M	C6	1	2	D	2	4	No	No	No
MWA	20 years	M	C6	5	4	A	0	0	Yes	Yes	No
MWA	31 years	M	C6	2	2	B	2	0	Yes	Yes	Yes
MWA	38 years	F	C5	3	2	B	3	0	No	Yes	No
MWA	53 years	M	C6	1	3	A	0	0	Yes	Yes	Yes
Fall	29 years	M	C4	4	4	A	0	0	Yes	Yes	Yes
MWA	28 years	F	C3	3	4		2		Yes	Yes	No
Bicycle accident	33 years	M	C6	3	4	A	0	0	Yes	No	No
Fall	54 years	F	CS	2	1	C	2	4	Yes	Yes	Yes
MWA	51 years	M	CS	1	1	D	2	2	No	No	No
Water slide accident	32 years	M	C6	3	4	A	0	0	Yes	No	No
Fall	65 years	F	C5	2	3	D	2	2	Yes	No	No
MWA	71 years	M	C6	1	2	D	2	3	No	No	No
MWA	23 years	M	C6	2	3	A	0	0	Yes	Yes	No
Fall	52 years	M	C4	1	0	D	4	4	No	No	No
Fall	22 years	M	C5	3	4	B	2	0	Yes	Yes	Yes
Fall	55 years	M	C5	5	4	A	0	0	Yes	Yes	Yes
Fall	15 years	F	C5	2	2	D	4	1	Yes	Yes	No
Surfing accident	60 years	F	C5	2	2	C	2	2	Yes	Yes	Yes
MWA	55 years	F	C5	2	2	D	4	3	No	No	No

Overview of included patients. *Trauma*, trauma mechanism that resulted in disability; *MWA*, motor vehicle accident; *Level*, level of injury in the cervical spine; *Sg. length*, sagittal extension of spinal cord damage in trauma MRI; *Basic*, basic score (0 to 4) by Talbott et al, grading transverse cord involvement; *A1S*, AIS grade (A to D), assessed according to ISNCSCI; *SEP*, SEP score (0 to 4); *MEP*, MEP score (0 to 4); *WD DC*, visible Wallerian degeneration in the dorsal column; *WD CS*, visible Wallerian degeneration in the lateral corticospinal tract; *WD ST*, visible Wallerian degeneration in the lateral spinothalamic tract

Among the 35 included patients, 26 were male, and 9 were female. Using the Kolmogorov-Smirnov test, age was normally distributed. Mean age was 44.3 years (SD ± 3.2), minimum 14.9 years, maximum 79.9 years. Cervical spine injury level ranged from C3 to C7. In 8.6% (*n* = 3), the level was C3; 22.9% (*n* = 8) had level C4; for 37.1% (*n* = 13), the level was at C5; 28.6% (*n* = 10) were injured at level C6; and 2.9% (*n* = 1) at level C7.

AIS A occurred in 34.3% (*n* = 12), AIS B in 17.1% (*n* = 6), C in 17.1% (*n* = 6), and AIS D in 31.4% (*n* = 11). The MEP score was distributed as follows: 0 points in 54.3% (*n* = 19), 1 point in 2.9% (*n* = 1), 2 points in 17.1% (*n* = 6), 3 points in 11.4% (*n* = 4), and 4 points in 14.3% (*n* = 5). SEP score was 0 points in 37.1% (*n* = 13), 1 point in 2.9% (*n* = 1), 2 points in 42.9% (*n* = 15), 3 points in 2.9% (*n* = 1), and 4 points in 14.3% (*n* = 5).

**Fig. 3:**
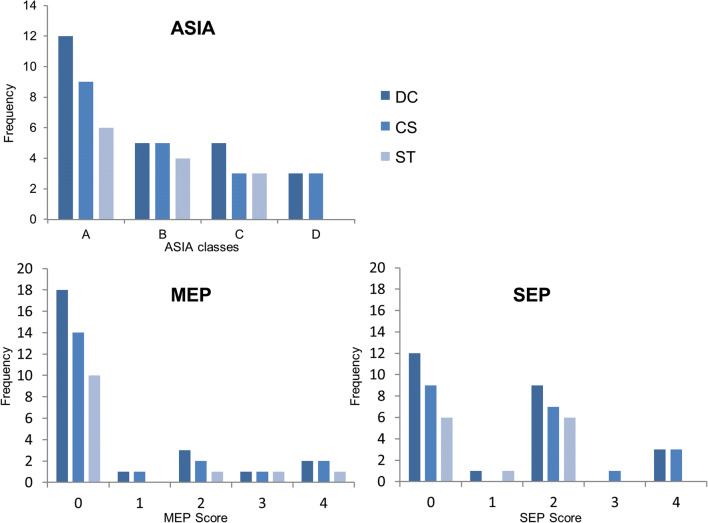
Distribution of observed WD among the different AIS grades as well as MEP and SEP scores. Out of 35 patients, signal intensity change in the DC was observed in an absolute number of 25, and in the CS and the ST in 20 and 13 cases, respectively. In cases with WD, this figure shows the distribution among the different AIS grades (A–D) and among the MEP and SEP scores (0–4 points)

Signal change in the spinal cord above the level of the injury in the dorsal column (DC) was visible in 71.4% (*n* = 25) and not visible in 28.6% (*n* = 10). Below the level of the injury, signal change in the lateral corticospinal tract (CS) could be observed in 57.1% (*n* = 20) and was not visible in 42.9% (*n* = 15). Signal change in the lateral spinothalamic tract (ST) above the level of the injury was visible in 37.1% (*n* = 13) and not visible in 62.9% (*n* = 22). Retrograde degeneration was not observed. Distribution of observed WD in the three different regions among the different AIS grades (A–D) and the different MEP and SEP scores (0–4) is given in Fig. 3.

**Fig. 4:**
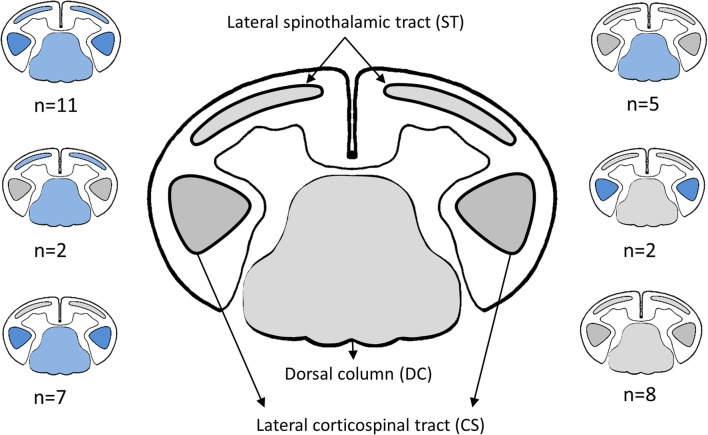
Cross-sectional drawing of the cervical cord and approximate anatomic location of the dorsal column (DC), the lateral corticospinal tract (CS), and the lateral spinothalamic tract (ST) of the cervical cord in a cross-sectional drawing. Smaller drawings indicate patterns of co-appearances of WD among the three tracts: Affected tracts are highlighted in blue. WD in the ST was only observed in combination with other tracts. Cross-sectional drawing of the cervical cord adapted from Nathan et al [17, 18]

In a considerable number of cases, signal change was present in more than one region. A display of co-appearances of signal change in the different anatomical regions is given in Fig. 4. When WD was visible, always the entire tract visible in the MRI examination did show WD (DC and ST above the lesion, CS below the lesion). We did not find affected and unaffected parts of the specific tracts in the same patients.

### Relation between typical spinal cord signal intensity change and clinical severity

Mann-Whitney *U* test was used to evaluate for differences in the presence or absence of WD in the different anatomical regions of the cervical cord in AIS grades as well as MEP and SEP scores. Results are given in Table 2.

**Table 2:** ASIA classes and MEP and SEP score in the presence or absence of WD

	ASIA			MEP			SEP		
	Median	*p*	*r*	Median	*p*	*r*	Median	*p*	*r*
DC	B vs D	< 0.001	0.62	0 vs 3	< 0.001	0.58	0 vs 2	0.031	0.36
CS	B vs D	0.016	0.41	0 vs 2	0.032	0.36	ns		
ST	B vs D	0.015	0.41	ns			ns		

Median of ASIA classes and MEP and SEP scores in the presence or absence of WD in the DC, PT, or ST. *p* value represents statistical significance, *r* value represents effect strength. For statistical analyses, ASIA classes were transferred to numeric (0–3), and results were transferred back to classes for reporting. *WD*, Wallerian degeneration; *MEP*, motor evoked potential; *SEP*, sensory evoked potential; *DC*, dorsal column; *CS*, lateral corticospinal tract; *ST*, lateral spinothalamic tract; *ns*, not significant

AIS grades were significantly different depending on the presence or absence of signal intensity change in the DC (*p* < 0.001). Median in the presence of hyperintense signal change was 1 (lower quartile 0.0; upper quartile 2.0), which is equivalent to AIS B; median in the absence of hyperintense signal change was 3 (2.75; 3.0), which is equivalent to AIS D. Calculation revealed a strong effect (*r* = 0.62). For CS, differences were significant (*p* = 0.016); median in the presence of hyperintense signal change was 1 (0.0; 2.0), equivalent to AIS B; without hyperintense signal intensity change, median was 3 (1.0; 3.0), equivalent to AIS D. Effect size was medium (*r* = 0.41). Significant differences were observed for the ST (*p* = 0.015) with an abnormal signal, median was 1 (0.0; 1.5) equivalent to AIS B. When the spinal cord appeared normal, median was 2.5 (0.0; 3.0) equivalent to AIS D. Effect size was medium (*r* = 0.41).

MEP scores were related to hyperintense signal change in the DC (*p* < 0.001), where WD was associated with a median score of 0 points (0.0; 1.5); median score with normal signal was 3 points (2.0; 4.0). Effect size was strong (*r* = 0.58). Association of MEP score with CS was significant (*p* = 0.032). Median score in the presence of hyperintense signal change was 0 points (0.0; 1.75); in the absence of hyperintense signal change, median was 2 points (0.0; 3.0). Effect size was medium (*r* = 0.36). Statistical significance was not reached for the ST (*p* = 0.067).

Evaluation of the SEP score reached statistical significance only for the dorsal column (*p* = 0.031); median was 1 point (0.0; 2.0) when signal was abnormal and 2 points (2.0; 3.25) when signal was normal. Effect size was moderate (*r* = 0.36). No correlation was found for SEP score with hyperintense signal change in CS but a trend for association of signal intensity change with ST (*p* = 0.092).

## Discussion

This study showed that Wallerian degeneration (WD) in the dorsal column (DC), the lateral corticospinal tract (CS), and in the lateral spinothalamic tract (ST) is commonly visible on T2-weighted MRI after traumatic cervical spine injury (tSCI). The presented data suggests a correlation between visible WD and clinical and neurophysiological impairment after trauma.

The concept of degeneration of an axon and its myelin sheath (or glia) distal to axonal injury was first described by Waller in 1850 after sectioning the glossopharyngeal nerve of frogs. It is well known in peripheral nerve injury and following spinal lesion; in contrast, the term “diaschisis” was introduced by von Monakow in 1914 representing a concept of focal neurological depression remote from the original site of damage, but anatomically connected by fiber tracts. Nowadays, crossed cerebellar diaschisis and WD revealed by MRI is the most widely known occurrence of this phenomenon [4].

After tSCI, tract-specific patterns of anterograde degeneration are found in histopathological preparations corresponding to signal alteration remote from the lesion in spinal MR imaging [2, 7, 17, 18]. Signal intensity change in the dorsal column on MRI has been shown as early as 7 weeks after injury [8]. Histopathologic changes could be observed as early as 8 days after injury without signal abnormality on MRI and showed the fasciculus gracilis above the lesion filled with axonal debris, whereas the fasciculus cuneatus was normal. As for the corticospinal tract below the injury, histopathological changes were visible after 12 days post injury while MRI changes could be observed after 7 weeks. Axonal debris was visible next to a normal spinocerebellar tract [8].

Advanced MRI techniques such as diffusion-weighted imaging (DWI) and diffusion tensor imaging (DTI) are used to depict early changes, attributed to acute WD [4, 11, 12, 14]. Other advanced techniques include magnetization transfer. Measurement of magnetization transfer ratios (MTR) in the dorsal spinal cord was able to predict sensory disability, whereas measures in the ventrolateral spinal cord predicted motor disability [9]. There is proof that tract alteration is not only an antegrade phenomenon since DTI technique has shown axonal degeneration parallel to the corticospinal tract above the level of the lesion and its association with disability [1]. In sum, there is increasing evidence on the value of advanced MRI techniques in providing prognostic information [4].

### Tract-specific Wallerian degeneration is commonly seen in routine MRI after tSCI

The presented data shows that appearance of tract-specific antegrade and retrograde degeneration, which we both refer to as WD, is a common phenomenon in routine MRI after severe tSCI. Especially WD in the DC remote from the injury can be seen in almost three quarters of the cases (71.4%) and can be appreciated with great confidence, represented by an absolute agreement between both readers. In the setting of a significant trauma history, misinterpretation is unlikely; still, mimics do exist: Deficiency syndromes, typically vitamin B_12_ [27, 28] can result in subacute combined degeneration (SACD) that can look identical. Different pathologies include demyelination, such as multiple sclerosis [29], but especially neuromyelitis optica because of its longitudinal cord extent [30] or inflammation such as in sarcoidosis [31].

Compared to the DC, identification of pathology in the CS or the ST was more difficult to detect, which is represented by an ICC of 0.54 and 0.88, respectively. Due to tract anatomy of the CS and the ST, there is only a small area of signal change on cross-sectional imaging, if WD is present. Unlike WD in the DC, pathology in the CS and ST was often visible as dot-like hyperintensities only. Signal intensity change reflecting WD in the CS could be observed in about one-half of the cases (57.1%). Mimics of this signal change include spinal ischemia, typically affecting the anterior spinal artery represented by the owl-eyes or snake-eyes sign [32], although in most cases, only few segments are involved. In this study, in the majority of the cases, WD of the CS occurred in combination with WD of the DC and ST. WD in the ST occurred only in combination with at least WD of the DC; in most cases, all three described types of WD were present. To our knowledge, no reasonable alternative exists for this specific pattern.

### Occurrence of Wallerian degeneration in the cervical cord is associated with clinical impairment

Analyses showed that appearance of WD in the three different locations is associated with a higher degree of clinical impairment, reflected by the AIS grades. Median AIS without visible degeneration was D, with degeneration, AIS was B.

MEP and SEP scores were different in the presence or absence of WD in the DC (0 vs 3 and 0 vs 2 points). MEP scores were also different in the presence or absence of WD in the CS (0 vs 2 points), while there was no significant difference for the SEP scoring. This could reflect the fact that the CS is only transmitting motor signals and a degeneration would not affect sensory transmission. Because of the overlap of WD in the different tracts, this conclusion is arguable and is also in contrast with high correlation of MEP score with WD in the DC. However, coincidental occurrence of WD in more than one tract would well be in line with a high likelihood of tSCI causing diffuse, extensive white matter damage in the spinal cord.

In line with these results, previous diffusion tensor imaging data in tSCI patients and correlation with clinical and electrophysiological measures suggested WD of spinal tracts remote from the injury site [14].

### Limitations

This study has the following limitations: although there is some evidence for tract-specific impairment, because of the large overlap between the three investigated regions, it was not possible to correlate tract degeneration with specific types (sensory/motor) of impairment more precisely. The visual localization and specification of spinal tracts is limited by low resolution and fibers of other origin are located in close proximity to, or interspersed with, the aforementioned tracts (for example fibers from the rubrospinal and reticulospinal tracts run anterolateral to the CS [18]). Hence, we acknowledge that the anatomical description used for the regions of interest should be understood as an approximation to real tract anatomy.

In this study, the onset of visible WD in routine MRI was highly variable; unfortunately, it was not possible to identify a typical time frame, when WD first occurs or to correlate its first occurrence with clinical features. Moreover, as imaging intervals between the included patients were unequal, it was not possible to systematically evaluate how the signal progresses over the time. Both questions may be addressed in a future, prospective study design.

### Summary statement

This study shows that WD especially in the dorsal column is a common phenomenon after severe spinal trauma, which can be detected by routine MRI with high confidence. Diagnosis in the appropriate clinical setting is straightforward. If Wallerian degeneration is present, it is associated with higher degree of clinical impairment.

## Abbreviations

AIS: American Spinal Injury Association Impairment Scale
CS: Lateral corticospinal tract
DC: Dorsal column
MEP: Motor evoked potential
SEP: Sensory evoked potential
ST: Lateral spinothalamic tract
tSCI: Traumatic spinal cord injury
WD: Wallerian degeneration

## References

[1] Freund P, Schneider T, Nagy Z (2012). Degeneration of the injured cervical cord is associated with remote changes in corticospinal tract integrity and upper limb impairment. PLoS One.

[2] Waller A (1850). Experiments on the section of the glossopharyngeal and hypoglossal nerves of the frog, and observations of the alterations produced thereby in the structure of their primitive fibres. Proc R Soc Lond.

[3] Kanamori A, Catrinescu MM, Belisle JM, Costantino S, Levin LA (2012) Retrograde and Wallerian axonal degeneration occur synchronously after retinal ganglion cell axotomy. Am J Pathol 181:62–73 10.1016/j.ajpath.2012.03.030 PMC3388161 22642911

[4] Chen YJ, Nabavizadeh SA, Vossough A, Kumar S, Loevner LA, Mohan S (2017) Wallerian degeneration beyond the corticospinal tracts: conventional and advanced MRI findings. J Neuroimaging 27:272–280 10.1111/jon.12404 28072502

[5] Valencia MP, Castillo M (2006). MRI findings in posttraumatic spinal cord Wallerian degeneration. Clin Imaging.

[6] Kuhn MJ, Mikulis DJ, Ayoub DM, Kosofsky BE, Davis KR, Taveras JM (1989) Wallerian degeneration after cerebral infarction: evaluation with sequential MR imaging. Radiology 172:172–182 10.1148/radiology.172.1.2740501 2740501

[7] Daniel PM, Strich SJ (1969). Histological observations on Wallerian degeneration in the spinal cord of the baboon, Papio papio. Acta Neuropathol.

[8] Becerra JL, Puckett WR, Hiester ED (1995). MR-pathologic comparisons of Wallerian degeneration in spinal cord injury. AJNR Am J Neuroradiol.

[9] Cohen-Adad J, El Mendili MM, Lehéricy S (2011). Demyelination and degeneration in the injured human spinal cord detected with diffusion and magnetization transfer MRI. Neuroimage.

[10] Huber E, Lachappelle P, Sutter R, Curt A, Freund P (2017) Are midsagittal tissue bridges predictive of outcome after cervical spinal cord injury? Ann Neurol 81:740–748 10.1002/ana.24932 28393423

[11] David G, Mohammadi S, Martin AR (2019). Traumatic and nontraumatic spinal cord injury: pathological insights from neuroimaging. Nat Rev Neurol.

[12] Shanmuganathan K, Zhuo J, Chen HH (2017). Diffusion tensor imaging parameter obtained during acute blunt cervical spinal cord injury in predicting long-term outcome. J Neurotrauma.

[13] Martin AR, De Leener B, Cohen-Adad J (2018). Monitoring for myelopathic progression with multiparametric quantitative MRI. PLoS One.

[14] Freund P, Seif M, Weiskopf N (2019). MRI in traumatic spinal cord injury: from clinical assessment to neuroimaging biomarkers. Lancet Neurol.

[15] Kashani H, Farb R, Kucharczyk W (2010). Magnetic resonance imaging demonstration of a single lesion causing Wallerian degeneration in ascending and descending tracts in the spinal cord. J Comput Assist Tomogr.

[16] Kirshblum S, Waring W (2014). Updates for the international standards for neurological classification of spinal cord injury. Phys Med Rehabil Clin N Am.

[17] Nathan PW, Smith MC, Deacon P (1990). The corticospinal tracts in man. Course and location of fibres at different segmental levels. Brain J Neurol.

[18] Nathan PW, Smith M, Deacon P (1996). Vestibulospinal, reticulospinal and descending propriospinal nerve fibres in man. Brain.

[19] Petersen JA, Wilm BJ, von Meyenburg J (2012). Chronic cervical spinal cord injury: DTI correlates with clinical and electrophysiological measures. J Neurotrauma.

[20] Talbott JF, Whetstone WD, Readdy WJ (2015). The brain and spinal injury center score: a novel, simple, and reproducible method for assessing the severity of acute cervical spinal cord injury with axial T2-weighted MRI findings. J Neurosurg Spine.

[21] Kirshblum SC, Biering-Sorensen F, Betz R (2014). International standards for neurological classification of spinal cord injury: cases with classification challenges. J Spinal Cord Med.

[22] Marino RJ, Barros T, Biering-Sorensen F (2003). International standards for neurological classification of spinal cord injury. J Spinal Cord Med.

[23] Deuschl G, Eisen A (1999) Long-latency reflexes following electrical nerve stimulation. The International Federation of Clinical Neurophysiology. Electroencephalogr Clin Neurophysiol Suppl 52:263–8 10590995

[24] Hupp M, Pavese C, Bachmann LM (2018). Electrophysiological multimodal assessments improve outcome prediction in traumatic cervical spinal cord injury. J Neurotrauma.

[25] Cohen J, Maydeu-Olivares A (1992). A power primer. Psychol Bull.

[26] Koo TK, Li MY (2016). A guideline of selecting and reporting intraclass correlation coefficients for reliability research. J Chiropr Med.

[27] Patil VM, Bhagwat KA, Gill HS, Khanapur R (2014). Sub-acute combined degeneration of the spinal cord - inverted ‘V’ sign a clue to avoid morbidity. Asian J Biomed Pharm Sci.

[28] Senol MG, Sonmez G, Ozdag F, Saracoglu M (2008). Reversible myelopathy with vitamin B12 deficiency. Singapore Med J.

[29] Lövblad KO, Anzalone N, Dörfler A (2010). MR imaging in multiple sclerosis: review and recommendations for current practice. Am J Neuroradiol.

[30] O’Riordan JI, Gallagher HL, Thompson AJ (1996). Clinical, CSF, and MRI findings in Devic’s neuromyelitis optica. J Neurol Neurosurg Psychiatry.

[31] Pawate S, Moses H, Sriram S (2009). Presentations and outcomes of neurosarcoidosis: a study of 54 cases. QJM.

[32] Novy J, Carruzzo A, Maeder P, Bogousslavsky J (2006). Spinal cord ischemia: clinical and imaging patterns, pathogenesis, and outcomes in 27 patients. Arch Neurol.

